# Upadacitinib-Induced Sepsis Resulting in Death in Crohn’s Disease: The First Reported Case

**DOI:** 10.5152/tjg.2026.25637

**Published:** 2026-01-30

**Authors:** Muhammed Furkan Keser, Yahya Atayan, Mustafa Duru, Ayşe Hafsa Çağın, Zeynep Büşra Keser, Yasir Furkan Çağın

**Affiliations:** 1 Department of Gastroenterology, İnonu University Faculty of Medicine, Malatya, Türkiye; 2 Department of Internal Medicine, Necmettin Erbakan University Faculty of Medicine, Konya, Türkiye; 3 Department of Infectious Diseases and Clinical Microbiology, İnonu University Faculty of Medicine, Malatya, Türkiye

Upadacitinib, a selective oral Janus kinase 1 (JAK1) inhibitor, is approved for the treatment of moderate-to-severe Crohn’s disease (CD).^1^ Sepsis and opportunistic infections associated with upadacitinib have been reported in patients with CD.^1^^,^^2^ This report presents the first documented fatal case of sepsis linked to a JAK1 inhibitor, underscoring its potential risk.

In the 20th month of follow-up, a 35-year-old male (62 kg, 175 cm; body mass index (BMI): 20.2) with refractory CD was started on upadacitinib 45 mg/day due to an inadequate response to adalimumab and azathioprine. He was classified as A2L1B2 per the Montreal classification, with a Crohn's Disease Activity Index (CDAI) score of 252.5. Initial assessment showed pitting edema, hypoalbuminemia (1.5 g/dL), anemia (Hb: 9.8 g/dL), and elevated C-reactive protein (CRP) (5.38 mg/dL). The patient had no known systemic or hematologic disease other than CD and was not receiving corticosteroids or any other immunosuppressive therapy at the time of upadacitinib initiation. Baseline hematologic evaluation showed normal leukocyte and platelet counts, with mild anemia consistent with chronic disease and no evidence of coagulopathy. Informed consent was obtained from the patient.

The therapy, initiated in the outpatient setting, was discontinued by the patient on day 4 due to fever. He presented on day 6 with worsening symptoms. On admission, he was febrile, confused, hypotensive, tachycardic, tachypneic, and had an oxygen saturation of 85%. Laboratory evaluation revealed profound neutropenia (0.44 × 10^3^/μL), leukopenia (total WBC 0.74 × 10^3^/μL), and thrombocytopenia (44 × 10^3^/μL). Coagulopathy was evident with an INR of 4.03, fibrinogen of 60 mg/dl accompanied by a D-dimer level (5.4 mg/L). Inflammatory markers were also high, with a CRP level of 10.5 mg/dL, supporting a severe infectious process. Biochemical analysis showed hypoalbuminemia (2.5 g/dL) and hyperbilirubinemia (total bilirubin 6.03 mg/dL, direct 3.31 mg/dL), consistent with liver dysfunction and disseminated intravascular coagulation (DIC). Based on the International Society on Thrombosis and Haemostasis (ISTH) overt DIC scoring system, the calculated score was 8, fulfilling the criteria for overt DIC.^3^ Blood, sputum, and urine cultures yielded no microbial growth, and no causative organism was identified. Viral polymerase chain reaction (PCR) assays from blood for CMV, EBV, and HSV, together with the corresponding IgM serologies, were all negative, thereby, excluding these specific viral infections. A respiratory multiplex PCR panel performed on nasopharyngeal and sputum samples, including SARS-CoV-2 testing, also detected no bacterial or viral pathogens. Bronchoscopy was not performed due to severe hemodynamic and respiratory instability after hospital admission. Cardiac causes and pulmonary embolism were excluded. Imaging revealed parenchymal consolidations consistent with pneumonia (Figure 1). Broad-spectrum antibiotics and G-CSF were initiated for pneumosepsis and secondary DIC. As hemodynamic instability persisted; vasopressors and mechanical ventilation were required. Despite all interventions, the patient died in the ICU.

Phase 3 trials have demonstrated upadacitinib’s efficacy in IBD. Approved by the FDA for TNF-refractory or intolerant Crohn’s and ulcerative colitis patients, it carries a boxed warning for serious infections.^1^^,^^4^^,^^5^ Severe infections and hematologic toxicities have been reported, especially during high-dose induction therapy.^1^ Associated adverse events include neutropenia, opportunistic infections (e.g., *P. jirovecii*, CMV, EBV), and bacterial complications such as anal abscess.^1^^,^^6^^,^^7^ In U-EXCEED, 1 patient developed neutropenia after 5 days of 45 mg upadacitinib, and treatment was stopped. A fatal infection occurred 5 months later but was not attributed to the drug.^1^

Neutropenia may develop in association with viral or bacterial infections, drug exposure, or nutritional deficiencies.^8^ In this patient, cultures showed no bacterial or fungal growth, and viral PCR assays, including blood, sputum, and respiratory multiplex testing for CMV, EBV, HSV, SARS-CoV-2, and other common respiratory viruses, were negative, excluding specific viral or fungal pathogens as the cause of neutropenia. Although hypoalbuminemia was present, normal BMI, absence of clinical or laboratory signs of malnutrition, normal vitamin levels, and lack of other drug exposure excluded nutritional or medication-related causes. However, because bronchoscopy could not be performed, lower respiratory pathogens such as *Pneumocystis jirovecii* could not be evaluated.

In our case, neutropenia during induction with upadacitinib led to rapidly progressive pneumosepsis and septic shock. The patient discontinued the drug after developing fever but delayed seeking medical care for more than 48 hours. Timely antibiotics reduce mortality in neutropenia; delays increase risk.^9^ Delayed presentation was the key determinant of the fatal outcome despite all interventions.

Pneumosepsis-induced DIC facilitated rapid progression to multi-organ dysfunction.

Although opportunistic infections associated with upadacitinib have been reported, this is the first documented case of septic shock and DIC resulting in death during CD treatment. The case highlights the fatal infectious risks of JAK1 inhibition and the need for prudent patient selection.

## Figures and Tables

**Figure 1.**
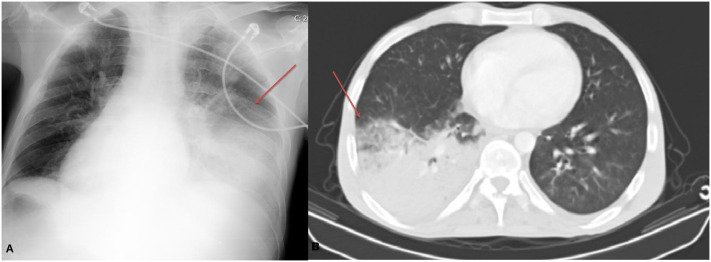
(A) Chest X-ray showing diffuse parenchymal opacities, more prominent in the right lower lung zone (arrow), consistent with consolidation. (B) Chest computed tomography (CT) image demonstrating dense consolidation with air bronchograms in the right lower lobe (arrow), compatible with pneumonia.

## Data Availability

The data that support the findings of this study are available on request from the corresponding author.

## References

[1] Loftus EV Jr Panés J Lacerda AP Upadacitinib induction and maintenance therapy for Crohn’s disease. N Engl J Med. 2023;388(21):1966 1980. (doi: 10.10.1056/NEJMoa2212728) 37224198

[2] Parra Izquierdo LV Frías-Ordoñez J Barreto Perez J P0915 Real-life experience with upadacitinib in Crohn’s disease (UPARECOL-CD induction): a Colombian cohort. J Crohns Colitis. 2025;19(suppl 1):i1721 i1722. (doi: 10.10.1093/ecco-jcc/jjae190.1089)

[3] Taylor FB Jr Toh CH Hoots WK Wada H Levi M Scientific Subcommittee on Disseminated Intravascular Coagulation (DIC) of the International Society on Thrombosis and Haemostasis (ISTH). Towards definition, clinical and laboratory criteria, and a scoring system for disseminated intravascular coagulation. Thromb Haemost. 2001;86(5):1327 1330. (doi: 10.10.1055/s-0037-1616068) 11816725

[4] Wu XP Lu XK Wang ZT Post-marketing safety concerns with upadacitinib: A disproportionality analysis of the FDA adverse event reporting system. Expert Opin Drug Saf. 2023;22(10):975 984. (doi: 10.10.1080/14740338.2023.2223952) 37310063

[5] Wang S Wang X Ding J Disproportionality analysis of upadacitinib-related adverse events in inflammatory bowel disease using the FDA adverse event reporting system. Front Pharmacol. 2025;16:1436183. (doi: 10.10.3389/fphar.2025.1436183) 40008128 PMC11851071

[6] Chin S Fox L Majumdar A Oliver M Choy MC De Cruz P. Pneumocystis jirovecii pneumonia complicating use of upadacitinib in a patient with ulcerative colitis and primary sclerosing cholangitis: a case report. Inflamm Bowel Dis. 2024;30(8):1435 1436. (doi: 10.10.1093/ibd/izae091) 38656421

[7] Richard N Amiot A Seksik P Effectiveness and safety of upadacitinib induction therapy for 223 patients with Crohn’s disease: a GETAID multicentre cohort study. Aliment Pharmacol Ther. 2025;61(10):1662 1670. (doi: 10.10.1111/apt.70073) 40038887 PMC12013792

[8] Newburger PE Dale DC. Evaluation and management of patients with isolated neutropenia. Semin Hematol. 2013;50(3):198 206. (doi: 10.10.1053/j.seminhematol.2013.06.010) 23953336 PMC3748385

[9] Rosa RG Goldani LZ. Cohort study of the impact of time to antibiotic administration on mortality in patients with febrile neutropenia. Antimicrob Agents Chemother. 2014;58(7):3799 3803. (doi: 10.10.1128/AAC.02561-14) 24752269 PMC4068526

